# The interplay mechanism between IDH mutation, MGMT-promoter methylation, and PRMT5 activity in the progression of grade 4 astrocytoma: unraveling the complex triad theory

**DOI:** 10.32604/or.2024.051112

**Published:** 2024-05-23

**Authors:** MAHER KURDI, ALAA ALKHOTANI, ABDULRAHMAN SABBAGH, EYAD FAIZO, AHMED I. LARY, AHMED K. BAMAGA, MAJID ALMANSOURI, BADR HAFIZ, THAMER ALSHARIF, SALEH BAEESA

**Affiliations:** 1 Department of Pathology, Faculty of Medicine, King Abdulaziz University, Rabigh, Saudi Arabia; 2 Department of Pathology, College of Medicine, Umm Al-Qura University, Makkah, Saudi Arabia; 3 Department of Surgery, Faculty of Medicine, King Abdulaziz University, Jeddah, Saudi Arabia; 4 Department of Surgery, Faculty of Medicine, University of Tabuk, Tabuk, Saudi Arabia; 5 Section of Neurosurgery, Department of Surgery, King Abdulaziz Medical City, Jeddah, Saudi Arabia; 6 Department of Pediatrics, Faculty of Medicine, King Abdulaziz University, Jeddah, Saudi Arabia; 7 Department of Clinical Biochemistry, Faculty of Medicine, King Abdulaziz University, Jeddah, Saudi Arabia; 8 Department of Neurosciences, King Faisal Specialist Hospital and Research Center, Jeddah, Saudi Arabia; 9 Department of Surgery, King Abdulaziz Specialist Hospital, Taif, Saudi Arabia

**Keywords:** Grade 4 astrocytoma, Glioblastoma, Isocitrate dehydrogenase (IDH), O-6-methylguanine-DNA methyltransferase (*MGMT)*, Protein methyltransferase proteins-5 (*PRMT5)*, Progression-free survival (PFS)

## Abstract

**Background:**

The dysregulation of Isocitrate dehydrogenase (IDH) and the subsequent production of 2-Hydroxyglutrate (2HG) may alter the expression of epigenetic proteins in Grade 4 astrocytoma. The interplay mechanism between IDH, O-6-methylguanine-DNA methyltransferase (*MGMT)*-promoter methylation, and protein methyltransferase proteins-5 (*PRMT5)* activity, with tumor progression has never been described.

**Methods:**

A retrospective cohort of 34 patients with G4 astrocytoma is classified into IDH-mutant and IDH-wildtype tumors. Both groups were tested for *MGMT*-promoter methylation and *PRMT5* through methylation-specific and gene expression PCR analysis. Inter-cohort statistical significance was evaluated.

**Results:**

Both IDH-mutant WHO grade 4 astrocytomas (n = 22, 64.7%) and IDH-wildtype glioblastomas (n = 12, 35.3%) had upregulated *PRMT5* gene expression except in one case. Out of the 22 IDH-mutant tumors, 10 (45.5%) tumors showed *MGMT*-promoter methylation and 12 (54.5%) tumors had unmethylated *MGMT*. All IDH-wildtype tumors had unmethylated *MGMT*. There was a statistically significant relationship between *MGMT*-promoter methylation and IDH in G4 astrocytoma (*p*-value = 0.006). Statistically significant differences in progression-free survival (PFS) were also observed among all G4 astrocytomas that expressed *PRMT5* and received either temozolomide (TMZ) or TMZ plus other chemotherapies, regardless of their IDH or *MGMT*-methylation status (*p*-value=0.0014). Specifically, IDH-mutant tumors that had upregulated *PRMT5* activity and *MGMT*-promoter methylation, who received only TMZ, have exhibited longer PFS.

**Conclusions:**

The relationship between *PRMT5*, *MGMT*-promoter, and IDH is not tri-directional. However, accumulation of D2-hydroxyglutarate (2-HG), which partially activates 2-OG-dependent deoxygenase, may not affect their activities. In IDH-wildtype glioblastomas, the 2HG-2OG pathway is typically inactive, leading to *PRMT5* upregulation. TMZ alone, compared to TMZ-plus, can increase PFS in upregulated *PRMT5* tumors. Thus, using a *PRMT5* inhibitor in G4 astrocytomas may help in tumor regression.

## Introduction

Grade 4 astrocytomas are malignant brain tumors that originate from neuroglial stem cells. It is subclassified by 2021-World Health Organization (WHO) and European Association of Neuro-Oncology (EANO) into isocitrate dehydrogenase (IDH)-mutant and IDH-wildtype tumors. IDH-wildtype tumors are isolated for glioblastomas [1,2]. There are currently no known risk factors for G4 astrocytoma aside from rare genetic mutations. The recommended initial treatment since 2005 is complete surgical removal followed by radiotherapy (RT) and temozolomide (TMZ) chemotherapy (CTx). However, in most cases, resistance to TMZ eventually develops [3]. The cause of this resistance is not yet determined. Despite this treatment, the median survival rate for G4 astrocytoma typically falls within the range of 14 to 15 months [3]. Targeting cellular pathways altered in G4 astrocytoma, such as mammalian target of rapamycin (*mTOR)*, epidermal growth factor receptor (*EGFR)* gene amplification, *PTEN* (phosphatase and tensin homolog) mutation and *TP53* mutation, have also failed to improve the outcome [4]. However, neither TMZ nor any other systemic adjuvants targeted these receptors have shown superiority over placebo in terms of efficacy [5]. Although there have been cases where certain additional therapies have provided extended periods of progression-free survival (PFS), no specific pharmacological intervention has demonstrated a significant efficacy in improving the overall course of the disease [1]. There is a growing interest in targeting not just the tumor cells but also the surrounding tumor microenvironment, including blood vessels, tumor-associated macrophages (TAMs), and tumor-infiltrating lymphocytes (TILs). *PD-L1* (programmed death-ligand 1) inhibitor targeting *PDL1* receptors on tumor microenvironment have also failed to prevent tumor progression [5–7].

**Figure 1:**
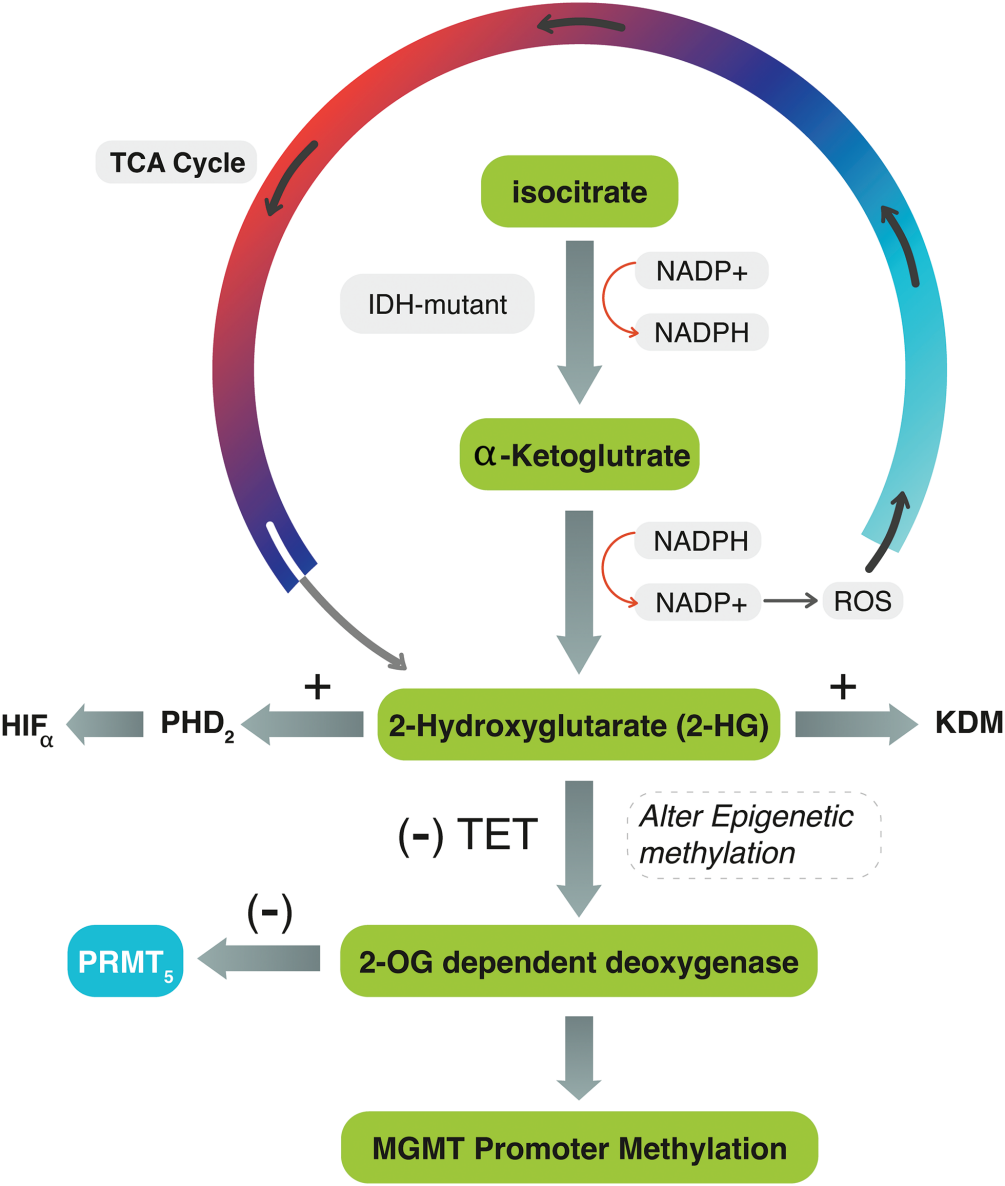
The diagram explains the interconnected mechanism between IDH, *MGMT*-promoter, and *PRMT5* genes during the TCA cycle. In IDH mutant tumors, IDH converts α-KG to 2-HG, activating ROS and PHD2 and generating NADP+. The accumulation of 2-HG stimulates HIF-α and inhibits TET enzymes, preventing the conversion of 5mC to 5hmC. It also competes with 2-OG for binding to the active site of 2-OG-dependent oxygenase. These reactions can lead to DNA methylation, chromatic modification, and impair PRMT5. In IDH-wildtype tumors, 2-HG is not produced, and PRMT5 may be upregulated.

Several biomarkers are still used in clinical practice to improve the diagnostic and prognostic significance of G4 astrocytomas. One of these biomarkers are IDH mutation and O-6-methylguanine-DNA methyltransferase (*MGMT)* gene promotor methylation. The IDH gene was shown to correlate with outcome of patients with G4 astrocytoma [8,9]. IDH is an enzyme encoded by IDH genes that catalyzes the oxidative decarboxylation of isocitrate to α-ketoglutarate (α-KG) in the tricarboxylic acid (TCA) cycle. In this reaction, IDH utilizes Nicotinamide Adenine Dinucleotide Phosphate (NADP+) as a cofactor and generates NADPH as a product [10]. The mutant isoforms of IDH catalyze the conversion of α-KG to the oncometabolite D2-hydroxyglutarate (2-HG) instead of α-KG, which inhibits α-KG-dependent enzymes, including histone demethylases, resulting in alterations of histone and DNA methylation [10,11] (Fig. 1).

The dysregulation of IDH and the subsequent production of 2-HG may affect the balance of NADPH and NADP+ levels in the cell. Consequently, the mutant IDH enzyme consumes NAD+ and generates NADH, leading to an increased NADH/NAD+ ratio. This altered ratio possesses various effects on cellular processes, including changes in energy metabolism and redox signaling, and stimulates the production of reactive oxygen species (ROS). Simultaneously, the accumulation of 2-HG within the cells increases the activity of prolyl hydroxylase domain 2 (PHD2), leading to the dysregulation (activation) of hypoxia-inducible factor-α (HIF-α). Oncometabolite 2-HG also inhibits the function of ten-eleven translocation (TET) enzymes, which are responsible for converting 5-methylcytosine (5mC) to 5-hydroxymethylcytosine (5hmC). It competes with 2-OG for binding to the active site of 2-OG-dependent oxygenase [11,12] (Fig. 1). All these effects may lead to a wide range of DNA methylation and chromatic modification and dysregulate epigenetic proteins such as protein methyltransferase proteins-5 (*PRMT5)*.

The effect of IDH products on the *MGM-*promoter and *PRMT5* was rarely investigated. *MGMT*, which is a DNA repair protein that removes alkyl groups from several residues, particularly the O6-position of guanine, is considered the most relevant for the action of TMZ [13,14]. The relationship between *MGMT-*promotor methylation and IDH mutation with treatment modalities and survival rates also showed controversial results [15]. Indeed, the potential predictive role of the *MGMT* status at recurrence needs to be further studied.

Protein arginine methylation (PRM) is the process of adding a methyl group to the amino acid arginine in proteins. This modification plays a crucial role in regulating various cellular processes. *PRMT5* is an epigenetic modifier that expressly methylates histones, thus exerting control over gene expression [16,17]. Upregulated or highly activated *PRMT5* expression has been linked to a poor prognosis in several types of cancer [18,19]. *PRMT5* gene expression varies in different grades of glioma: low in low-grade gliomas and high in malignant gliomas [20]. This is because most low-grade gliomas may have mutant-IDH. IDH-mutant WHO grade-4 astrocytomas that cause excessive production of 2-HG may also alter 2-OG-dependent dioxygenase enzymes, eventually leading to *PRMT5* inhibition [21] (Fig. 1). This effect would decrease the likelihood of tumor cell proliferation and progression. Suva et al. found that IDH-mutant WHO G4 astrocytomas exhibit lower levels of *PRMT5* expression compared to IDH-wildtype glioblastoma, suggesting that *PRMT5* may be influenced by IDH mutation [21]. In IDH-wildtype glioblastoma, the production of 2-HG is absent, allowing for *PRMT5* evolution. Kurdi et al. also observed this pattern in their study conducted in 2024, where upregulated *PRMT5* in brain tumor tissues was associated with earlier tumor recurrence in IDH-wildtype glioblastomas [22].

Our research study aims to explore the impact of IDH mutation, both at the mutational and non-mutational level, on *MGMT*-promoter methylation and *PRMT5* gene expression. Additionally, it will assess the effect of IDH and *MGMT*-promoter on PFS in tumors with upregulated *PRMT5*. The findings of this study will deliberate the complex triad theory beyond these three determinants.

## Materials and Methods

### Patients stratification

**Figure 2:**
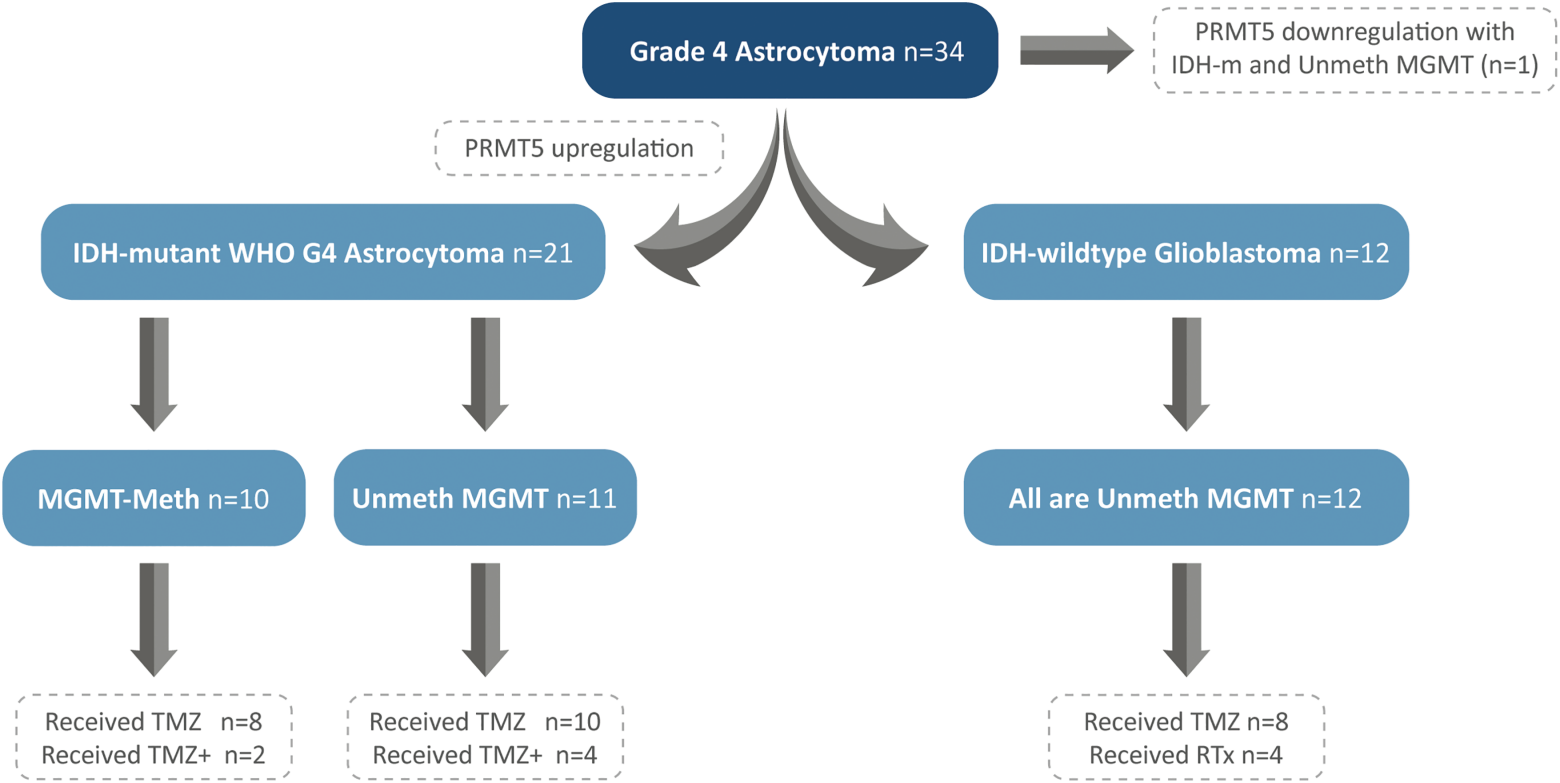
Patients’ stratification into IDH-mutant WHO-Grade 4 astrocytomas and IDH-wildtype glioblastomas, with segregation of *MGMT*-promoter and *PRMT5* gene expression.

The study included 34 patients who were diagnosed with Grade 4 astrocytoma and underwent complete surgical resection at a single medical institution in Saudi Arabia between 2018 and 2020. Patients’ written informed consents have been previously obtained. The study was approved by the Biomedical Ethics Committee between King Faisal Specialist Hospital and King Abdulaziz University under a decree of (HA-02-J-008). The histopathological diagnosis followed the 2021-WHO classification of central nervous system (CNS) tumors [1]. Patients’ clinical data, including IDH-mutation status, treatment protocol, and PFS, were obtained from hospital records. The patients were classified into two groups: IDH-mutant and IDH-wildtype tumors. Both groups were tested for *MGMT*-promoter methylation and *PRMT5* gene expression (Fig. 2). A 4-µm formalin-fixed and paraffin-embedded (FFPE) tissue sections were used during tissue processing for DNA and RNA extractions.

### Tissue processing

#### DNA extraction and MGMT-promoter methylation sequencing

All samples in our study have been retrospectively tested for *MGMT*-promoter methylation using a qualitative methylation-specific polymerase chain reaction (MS PCR) to detect *MGMT*-promoter methylation. The MSP assay is utilized to detect CpG island methylation with high sensitivity and specificity. H&E-stained sections of 34 blocks were examined to select regions with a high content of tumor cells. DNA extraction was performed using the QIAamp DNA FFPE tissue Kit from Qiagen (Venlo, Netherlands) following the manufacturer’s instructions. The quantity and quality of the extracted DNA were assessed using a nanodrop spectrophotometer at A250/A260 and A240/A230 ratios. The concentration of each DNA sample was standardized to 45 ng and subsequently subjected to bisulfate treatment using the EpiTect bisulfited Kit from Qiagen (Maryland, USA). Qualitative detection of *MGMT*-promoter methylation was performed using MSP, as described by Esteller et al. [23]. The forward and reverse primers targeting methylated and unmethylated exon 1 of the human *MGMT*-promoter gene are described in Table 1.

**Table 1:** Clinical and biological information of the 34 patients with G4 astrocytoma

#	IDH status	PRMT5 expression	MGMT methylation	Treatment	CTx	PFS
**1**	IDH-m	Upregulated	Methylated	RT+CTx	TMZ	747
**2**	IDH-m	Upregulated	Methylated	RT+CTx	TMZ	359
**3**	IDH-m	Upregulated	Methylated	RT+CTx	TMZ	566
**4**	IDH-m	Upregulated	Methylated	RT+CTx	TMZ	339
**5**	IDH-m	Upregulated	Methylated	RT+CTx	TMZ+	684
**6**	IDH-m	Upregulated	Methylated	RT+CTx	TMZ	461
**7**	IDH-m	Upregulated	Methylated	RT+CTx	TMZ+	549
**8**	IDH-m	Upregulated	Methylated	RT+CTx	TMZ	723
**9**	IDH-m	Upregulated	Methylated	RT+CTx	TMZ	210
**10**	IDH-m	Upregulated	Methylated	RT+CTx	TMZ	1100
**11**	IDH-m	Upregulated	Unmethylated	RT+CTx	TMZ	600
**12**	IDH-m	Downregulated	Unmethylated	RT+CTx	TMZ+	1400
**13**	IDH-m	Upregulated	Unmethylated	RT+CTx	TMZ	177
**14**	IDH-m	Upregulated	Unmethylated	RT+CTx	TMZ	350
**15**	IDH-m	Upregulated	Unmethylated	RT+CTx	TMZ	293
**16**	IDH-m	Upregulated	Unmethylated	RT+CTx	TMZ	623
**17**	IDH-m	Upregulated	Unmethylated	RT+CTx	TMZ	485
**18**	IDH-m	Upregulated	Unmethylated	RT+CTx	TMZ	550
**19**	IDH-m	Upregulated	Unmethylated	RT	None	155
**20**	IDH-m	Upregulated	Unmethylated	RT+CTx	TMZ	762
**21**	IDH-m	Upregulated	Unmethylated	RT+CTx	TMZ	548
**22**	IDH-m	Upregulated	Unmethylated	RT+CTx	TMZ	229
**23**	IDH-w	Upregulated	Unmethylated	RT+CTx	TMZ	198
**24**	IDH-w	Upregulated	Unmethylated	RT+CTx	TMZ	191
**25**	IDH-w	Upregulated	Unmethylated	RT	None	217
**26**	IDH-w	Upregulated	Unmethylated	RT+CTx	TMZ	180
**27**	IDH-w	Upregulated	Unmethylated	RT+CTx	TMZ	430
**28**	IDH-w	Upregulated	Unmethylated	RT+CTx	TMZ	190
**29**	IDH-w	Upregulated	Unmethylated	RT+CTx	TMZ	211
**30**	IDH-w	Upregulated	Unmethylated	RT	None	1128
**31**	IDH-w	Upregulated	Unmethylated	RT	None	80
**32**	IDH-w	Upregulated	Unmethylated	RT+CTx	TMZ	208
**33**	IDH-w	Upregulated	Unmethylated	RT	None	455
**34**	IDH-w	Upregulated	Unmethylated	RT+CTx	TMZ	433

The PCR Kit used in this study was the HotStarTaq plus DNA polymerase from Qiagen (Maryland, USA). The thermal cycling process was performed on a Veriti thermal cycler (Thermo-Fisher, Waltham, Massachusetts, USA). The cycling protocol consisted of an initial step at 95°C for two minutes, followed by 45 cycles of 35 s at 95°C, 30 s at 50°C, and 25 s at 70°C for 10 min. Both methylated and non-methylated control DNA samples were included in each run to ensure optimum sample accuracy. The PCR products were then visualized using 8% non-denaturing polyacrylamide gels and stained with ethidium bromide. Samples that showed amplification of a methylated sequence were considered methylation positive. The cut-off value which determined the percentage of methylated amplicons detected in an unmethylated control was used to distinguish between unmethylated and methylated tumors. The methylation results are summarized in Table 1.

#### RNA extraction, cDNA synthesis, and PRMT5 Gene expression assay using qPCR

RNA was extracted from 34 FFPE tissue blocks using the Qiagen Kit (Venlo, Netherlands) following the instructions provided by the manufacturer. The extraction process involved centrifugation and deparaffinization with xylene, followed by multiple washes with ethanol to ensure the cleanliness of the samples. After drying, a mixture of 150 µL Proteinase K Digestion Buffer (PKDB) and 10 µL Proteinase K (PK) was prepared and incubated at different temperatures (50°C, 75°C, and on ice). A combination of 300 µL RBC lysing buffer and ethanol was vortexed, centrifuged, and discarded. The resulting dry spin column was then transferred to a free-RNase tube. From the eluates containing RNA, a suitable sample was selected for spectrophotometric analysis. To generate complementary DNA (cDNA), the Reverse Transcription (RT) Kit (Applied Biosystems™, California, USA) was utilized. The master mix was prepared using RT buffer, dNTP mix, and random primers. The RT mixture was combined with 45ng of RNA, and the final volume was adjusted to 12 µL using RNase-free water. The primers for the experimental gene (*PRMT5*) and two reference genes (*GAPDH* and *ACTB*) were designed. The *PRMT5* primers used for qPCR sequences (Haven Scientific, Thuwal, KSA) are described by Kurdi et al. [22] (Table 2).

**Table 2:** Primers used for testing *MGMT*-promoter methylation and *PRMT5* gene expression

Primer	Sequence
**MGMT-Promoter gene**	
MSP-MGMT-Meth-Forward	5′-TTTCGACGTTCGTAGGTTTTCGC-3′
MSP-MGMT-Meth-Reverse	5′-GCACTCTTCCGAAAACGAAACG-3′
MSP-MGMT-UnMeth-Forward	5′-TTTGTGTTTTGATGTTTGTAGGTTTTTGT-3′
MSP-MGMT-UnMeth-Reverse	5′-AACTCCACACTCTTCCAAAAACAAAACA-3′
**PRMT-5 gene**	
PRMT5-Forward	5′- GAGTATCCGTCCAGAGACTCAC -3′
PRMT5-Reverse	5′- ACCGTTATGGGCTGCTTAATAG -3′

RT-qPCR was performed using the EverGreen Universal qPCR Master Mix (Haven Scientific, Thuwal, KSA). The synthesized cDNA was combined with the EverGreen master mix and a small amount of each oligo to create the final PCR reaction. Triplicate reactions were performed to ensure sample accuracy. The plates were sealed with the RT-qPCR adhesive seal. For analysis, two replicates of threshold cycle (CT) values were obtained for both the target gene (*PRMT5*) and the reference genes (*GAPDH* and *ACTB*). The relative quantification (Rq) and fold change (FC) were estimated to measure gene expression. These values provide insights into the expression levels of the genes. The results of these data analysis can be found in the supplementary file (Suppl. Table S1) attached to this article. however, gene expression results are summarized in Table 1.

#### Statistical interpretation

Data are described as frequencies and percentages. PFS is the time estimated after surgical resection to the first day of tumor recurrence. Pearson’s Chi-Square test was used to explore the association between *MGMT*-promoter and IDH in all *PRMT5* expressed or non-expressed tumors. The Kaplan Meier curves (KMC) and log-rank tests were used to compare the distribution of PFS across all different groups. All statistical analyses were performed using IBM SPSS1 ver. 24 statistical software (IBM Corp., Armonk, NY, USA).

## Results

Patients are subclassified into two groups: group (a) IDH-mutant WHO grade 4 astrocytoma (n = 22, 64.7%), and group (b) IDH-wildtype glioblastoma (n = 12, 35.3%) (Fig. 2). All tumors showed *PRMT5* gene upregulation (n = 33) except in one case (Fig. 2) (Table 1). Of the 22 IDH-mutant tumors, 10 (45.5%) tumors had *MGMT*-promoter methylation, and 12 (54.5%) tumors had unmethylated *MGMT-*promoter. All IDH-wildtype tumors (n = 12) had unmethylated *MGMT*. There was a statistically significant relationship between *MGMT*-promoter methylation and IDH in G4 astrocytoma (*p*-value = 0.006) (Table 3).

**Table 3:** The relationship between MGMT- methylation and IDH in G4 astrocytomas

		IDH-mutant	IDH-wildtype	Total	*p*-value
		22 (64.7)	12 (35.3)	34	
**MGMT-methylation**	Methylated	10 (45.5)	0 (0.0)	10 (29.4)	0.006
	Unmethylated	12 (54.5)	12 (100.0)	24 (70.6)	

**Figure 3:**
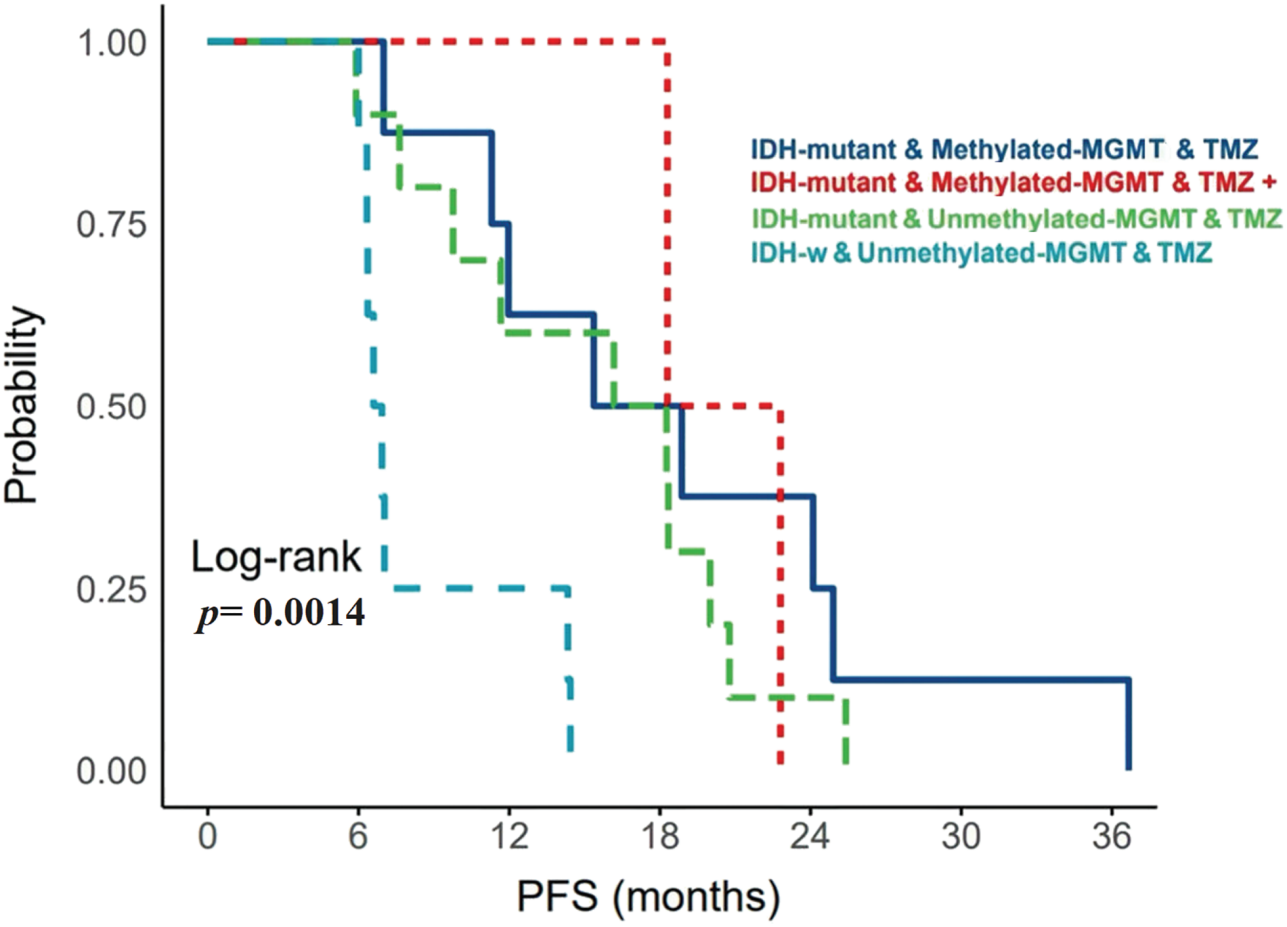
The relationship between *MGMT*-promoter and upregulated *PRMT5* gene expression with PFS in patients with IDH-mutant WHO G4 astrocytoma and IDH-wildtype glioblastoma. *MGMT: O-6-methylguanine-DNA methyltransferase; TMZ: Temozolomide; W: Wildtype*.

In total, 29 patients received a standard protocol of RT and CTx after surgery, while 4 patients received only RT. Patients who received CTx have been stratified based on IDH mutation and *MGMT*-promoter methylation (Fig. 2). They either received TMZ therapy or TMZ plus other chemotherapeutic agents such as chloroethylating agent, procarbazine, and/or bevacizumab. Standard RT was given as a total dose of 60 Gy, and the TMZ was given at 150–200 mg/m^2^ for 5 days every 28 days for 6–12 cycles. Statistically significant differences in PFS were observed among all G4 astrocytomas that expressed *PRMT5* and received either TMZ or TMZ plus other chemotherapies, regardless of their IDH or *MGMT*-promoter methylation status (*p*-value = 0.0014) (Fig. 3).

Specifically, IDH-mutant tumors that had upregulated *PRMT5* and *MGMT*-promoter methylation, who received only TMZ, have exhibited longer PFS. On the other hand, IDH-wildtype glioblastoma cases that expressed *PRMT5* and had unmethylated *MGMT*-promoter, who received TMZ treatment, have experienced early tumor recurrence.

## Discussion

The end-product of isocitrate oxidative decarboxylation into α-KG by the IDH enzyme in the TCA cycle typically determines the action of protein methylation in tumor cells. When the IDH gene mutates, the metabolic product ends with 2-HG, which inhibits histone demethylases, disrupts HIF-α regulation, and alters epigenetic proteins through the 2-OG-dependent dioxygenase pathway. This alteration can potentially deactivate the DNA-repair function of *MGMT* and inhibit the *PRMT5* gene expression [10]. On the other hand, if the IDH gene does not mutate, the oxidative decarboxylation process is interrupted, which may result in the failure to methylate *MGMT-*promoter. Consequently, *PRMT5* may be upregulated (Fig. 1). Although this proposed theory has not been described before, the logical relationship between these determinants explains their association. The association between IDH-wildtype glioblastomas and *PRMT5* upregulation has been previously reported by Kurdi et al. and Suva et al. [21,22]. The higher the grade, the more aggressive the astrocytoma with wildtype IDH, and the more significant the upregulation of *PRMT5*. However, this relationship does not apply to all cases of G4 astrocytoma with IDH mutation. Our current research provides a clear example, where 50% (n = 11/22) of IDH-mutant G4 astrocytoma showed upregulated *PRMT5* expression, and 10 of them had unmethylated *MGMT-*promoter. This indicates that mutant isoforms of IDH may not directly deactivate the DNA repair function of *MGMT* or inhibit *PRMT5*. Other unidentified or non-specific gene drivers or demethylases could potentially be involved in maintaining the expression of *PRMT5* or in demethylating the *MGMT*-promoter region.

Although the sensitivity of *MGMT*-methylated tumors to chemotherapeutic agents varies, *MGMT*-promoter is considered the most relevant for the action of TMZ [13,14]. Its relationship with G4 astrocytoma treatment modalities and clinical outcomes showed provocative results [15]. According to various studies, there is conflicting evidence regarding the sensitivity of G4 astrocytomas with methylated *MGMT*-promoter to TMZ treatment. Some studies suggest that G4 astrocytomas are sensitive to TMZ, while others indicate that *MGMT*-promoter methylation is not associated with a favorable outcome [23–26]. Despite this controversy, *MGMT*-promoter methylation is still considered a prognostic factor for patients with G4 astrocytoma. Kurdi et al. found that using TMZ or TMZ plus other chemotherapeutic agents did not result in significant differences in PFS for G4 astrocytomas, regardless of their IDH status [15]. However, the same study suggested that IDH-wildtype glioblastomas with unmethylated *MGMT*-promoter may benefit from combined chemotherapeutic modalities to improve prognosis. *PRMT5* gene dysregulation can occur in IDH-mutant WHO-G4 astrocytomas and IDH-wildtype glioblastomas, regardless of *MGMT*-promoter status. However, inhibiting the *PRMT5* activity may assist in suppressing tumor growth in both cases. Kurdi et al. briefly described how blocking the *PRMT5* receptor can lead to regression of tumor cells [22]. It is worth noting that downregulation of *PRMT5* in IDH-mutant WHO G4 astrocytomas or IDH-wildtype glioblastomas is associated with a more favorable prognosis.

There is evidence to suggest that the growth of glioma may be reliant on *PRMT5* expression, making it a potential therapeutic target [27]. Otani et al. demonstrated that blocking *PRMT5* activity led to apoptosis in stem-like glioma cells and restoration of immune response [28]. Abe et al. suggested that *PRMT5* plays a role in promoting the immunosuppressive characteristics of the tumor microenvironment [29]. Consequently, inhibiting *PRMT5* may contribute to the recovery of the immune cycle and enhance the immune system’s ability to combat cancer cells. Preclinical studies, *in vitro* and *in vivo*, revealed that among the *PRMT5* inhibitors, treatment of glioblastoma with compound 5 (CMP5) reflects the impact of *PRMT5* knockdown, which results in apoptosis of differentiated cells driven into a nonreplicated aging state [27]. *PRMT5* inhibitor has also been shown to sensitize glioma cells to radiation and chemotherapies [27,30,31]. This suggests that combining a *PRMT5* inhibitor with existing chemotherapies may prevent tumor progression. There is no clear evidence on whether IDH mutation or *MGMT* promoter methylation can affect the anti-tumor activity of *PRMT5* in G4 astrocytoma when used in conjunction with other chemotherapeutic agents. However, it has been suggested that a *PRMT5* inhibitor may increase the sensitivity of glioma cells to chemotherapies. It is unclear which chemotherapies interact more effectively with a *PRMT5* inhibitor. Nevertheless, using multiple regimen chemotherapies can potentially enhance patients’ survival by inhibiting tumor re-growth in different ways [15].

In our current study, we have observed that there were significant differences in PFS of all G4 astrocytoma with upregulated *PRMT5*, receiving TMZ or TMZ plus other chemotherapeutic agents, regardless of IDH or *MGMT*-promoter status (Fig. 3). IDH-mutant WHO-G4 astrocytomas with upregulated *PRMT5* and methylated *MGMT*-promoter who received only TMZ (n = 8) had the longest PFS. Additionally, IDH-mutant tumors regardless of *MGMT*-promoter status, who received only TMZ had better PFS than those who received TMZ plus other chemotherapies (Fig. 3). Our findings are interesting but might not be satisfactory because of the low number of samples acknowledged in our research.

Herein, two possible observations are noticed. First, our findings are consistent with previously mentioned theories emphasizing that 2-HG end-product of mutant IDH alters the epigenetic proteins in the DNA of these tumors. This alteration should frequently cause *MGMT*-methylation and *PRMT5* inhibition. However, we found that 2-HG in most IDH-mutant cases caused partial methylation of *MGMT*-promoter but upregulation of the *PRMT5* gene in 97% of the tumors. This probably occurred because 2-HG causes partial activation of 2-OG-dependent dioxygenases. There is no clear evidence beyond this finding. However, possible unknown factors may have participated in this mechanism of action. One of the potential theories is HIF-α. HIF-α is activated by 2-HG, a product of IDH-mutation, which indirectly affects 2-OG-dependent dioxygenase under hypoxic conditions. 2-OG is highly sensitive to oxygen, and when the oxygen level is high, HIF-α dysregulates and activates 2-OG [32]. Moreover, a lack of vitamin C levels in the body may also indirectly activate 2-OG [33].

Second, IDH-mutant tumors with upregulated *PRMT5* activity responded well to TMZ than TMZ plus other chemotherapies, which subsequently improved PFS (Fig. 3). Nonetheless, using TMZ with other chemotherapies may decrease the sensitivity of the tumor cells to TMZ, regardless of *MGMT*-promoter status. *MGMT*-methylation has no substantial effect on tumor response to CTx [15]. It is unclear if one of these chemotherapies used has reduced the effect of TMZ or the sensitivity of tumor cells to TMZ had less response. This theory is mainly based on IDH-mutant WHO-G4 astrocytoma. In IDH-wildtype glioblastomas, using multiple regimes of chemotherapies regardless of *MGMT*-promoter status is better than using single TMZ therapy, as Kurdi et al mentioned in their study [15]. In our current study, the theory was not clearly defined as all IDH-wildtype tumors with unmethylated *MGMT*-promoter and increased *PRMT5* activity were treated solely with TMZ, resulting in a low PFS. Additionally, we hypothesize that the efficacy of TMZ in G4 astrocytoma may improve with the use of *PRMT5* inhibitors, irrespective of the methylation status of *MGMT*-promoter or IDH mutational status.

## Conclusion

The relationship between *PRMT5* activity and *MGMT*-promoter methylation with IDH mutation is not tri-directional. Partial activation of 2-OG-dependent deoxygenase due to the accumulation of 2-HG in IDH-mutant tumors may not have an impact on *MGMT* or *PRMT5* activity. In IDH-wildtype glioblastomas, the 2HG-2OG pathway remains inactive, leading to persistent upregulation of *PRMT5*. TMZ alone has been observed to increase PFS in upregulated *PRMT5* tumors. However, the use of TMZ with other chemotherapies may impede tumor regression for reasons that are currently unknown. *PRMT5* inhibitors in both isoforms may help in tumor regression.

## Data Availability

The datasets generated during and/or analysed during the current study are available from the corresponding author (MK) on reasonable request. Dataset is represented in a supplementary file.

## Supplementary Materials

**Table S1:** Raw data results of CT-value and gene expression used in the RT-PCR processing of all patients’ tissues involved in the study. This table is supplementary to Table 1

#	ACTB Ct	GAPDH Ct	PRMT5 Ct	PRMT5 expression
1	26.712	26.640	28.887	Upregulated
2	32.656	29.961	35.013	Upregulated
3	30.684	27.271	34.997	Upregulated
4	31.195	25.958	33.975	Upregulated
5	31.938	27.298	33.867	Upregulated
6	30.803	26.479	31.698	Upregulated
7	30.495	28.228	32.208	Upregulated
8	33.450	31.486	35.619	Upregulated
9	31.018	26.501	32.544	Upregulated
10	33.198	27.299	33.911	Upregulated
11	35.828	32.589	37.702	Upregulated
12	33.230	29.435	34.878	Downregulated
13	33.592	29.404	35.475	Upregulated
14	31.415	27.427	32.274	Upregulated
15	31.823	28.840	33.469	Upregulated
16	30.803	26.479	31.698	Upregulated
17	31.341	28.673	32.756	Upregulated
18	31.431	28.680	32.768	Upregulated
19	32.795	30.107	33.256	Upregulated
20	35.779	32.587	38.387	Upregulated
21	32.088	28.061	33.008	Upregulated
22	31.551	27.116	31.643	Upregulated
23	34.666	26.032	30.802	Upregulated
24	32.203	27.570	31.932	Upregulated
25	33.163	29.843	33.600	Upregulated
26	30.643	26.032	30.802	Upregulated
27	32.231	26.251	31.319	Upregulated
28	35.779	32.587	38.387	Upregulated
29	30.777	26.633	30.588	Upregulated
30	31.693	26.498	32.022	Upregulated
31	31.946	27.113	31.079	Upregulated
32	34.794	31.737	34.256	Upregulated
33	33.875	30.914	34.345	Upregulated
34	31.205	25.597	31.226	Upregulated
